# Effect of automated head-thorax elevation during chest compressions on lung ventilation: a model study

**DOI:** 10.1038/s41598-023-47727-z

**Published:** 2023-11-21

**Authors:** Hélène Duhem, Nicolas Terzi, Nicolas Segond, Alexandre Bellier, Caroline Sanchez, Bruno Louis, Guillaume Debaty, Claude Guérin

**Affiliations:** 1 SAMU 38, Centre Hospitalier Universitaire Grenoble Alpes, 38043 Grenoble, France; 2 Université de Grenoble-Alpes/CNRS, UMR 5525Univ. Grenoble Alpes, CNRS, UMR 5525, VetAgro Sup, Grenoble INP, TIMC, 38000 Grenoble, France; 3 Médecine Intensive Réanimation, Centre Hospitalier Universitaire Grenoble Alpes, 38043 Grenoble, France; 4 Institut Mondor de Recherches Biomédicales INSERM-UPEC UMR 955 Eq13 - CNRS EMR 7000, 8 rue du Général Sarrail, 94010 Créteil, France; 5 Faculté de médecine Lyon Est, Université de Lyon, 8 avenue Rockefeller, 69373 Lyon cedex 08, France

**Keywords:** Respiration, Preclinical research

## Abstract

Our goal was to investigate the effects of head-thorax elevation (HUP) during chest compressions (CC) on lung ventilation. A prospective study was performed on seven human cadavers. Chest was automatically compressed-decompressed in flat position and during progressive HUP from 18 to 35°. Lung ventilation was measured with electrical impedance tomography. In each cadaver, 5 sequences were randomly performed: one without CC at positive end-expiratory pressure (PEEP) 0cmH_2_O, 3 s with CC at PEEP0, 5 or 10cmH_2_O and 1 with CC and an impedance threshold device at PEEP0cmH_2_O. The minimal-to-maximal change in impedance (VT_EIT_ in arbitrary unit a.u.) and the minimal impedance in every breathing cycle (EELI) the) were compared between flat, 18°, and 35° in each sequence by a mixed-effects model. Values are expressed as median (1st–3rd quartiles). With CC, between flat, 18° and 35° VT_EIT_ decreased at each level of PEEP. It was 12416a.u. (10,689; 14,442), 11,239 (7667; 13,292), and 6457 (4631; 9516), respectively, at PEEP0. The same was true with the impedance threshold device. EELI/VT_EIT_ significantly decreased from − 0.30 (− 0.40; − 0.15) before to − 1.13 (− 1.70; − 0.61) after the CC (P = 0.009). With HUP lung ventilation decreased with CC as compared to flat position. CC are associated with decreased in EELI.

## Introduction

Standard cardiopulmonary resuscitation (CPR) includes chest compressions and mechanical/manual ventilation aiming at restoring spontaneous circulation and maintaining gas exchange. The level of evidence for ventilator settings during CPR is weak^1^, and our understanding of lung physiology during CPR is limited. During chest compressions, intrathoracic pressure increases, and end-expiratory lung volume (EELV) decreases below the closing volume leading to intra-thoracic airway closure^2^ and atelectasis^3^. During chest decompressions the increase in chest wall elastic recoil promotes venous return, thereby improving hemodynamics, and possibly favors inspiratory flow due to the negative pressure in the thorax and intra-thoracic airways. However, in the presence of airway closure the negative airway pressure (Paw) may not be transmitted to the airway opening preventing the inflow of air towards the alveoli^4^. Low levels of positive end-expiratory airway pressure (PEEP) can maintain airway patency and inspiratory flow^4^ but, on the other hand, PEEP may impair venous return, right ventricle function and cerebral perfusion. An impedance threshold device (ITD) that induces negative intra-thoracic and increases venous return improves coronary blood flow and energy requirement during a porcine model of ventricular fibrillation model^5^. Combination of ITD with active compression-decompression CPR improves survival with favorable neurologic outcome^6^.

Lung protection becomes a priority whenever mechanical ventilation is applied. This may also be the case during CPR, notwithstanding the above considerations regarding hemodynamic efficiency and gas exchange preservation that are of utmost importance in the setting of cardiac arrest^7–9^. Low PEEP during CPR should contribute to lung protection as do low tidal volume and a low respiratory rate^1^. Automated controlled sequential elevation of the head and thorax inclination (HUP) during CPR has been shown to improve cerebral perfusion in animal models^10,11^ and may improve outcome in patients^12^. Moreover, HUP in conjunction with ITD can improve neurological outcome in animal model of cardiac arrest^13^. Since this maneuver can potentially stabilize EELV and minimize the large swings in lung volume during CPR, it may benefit both the hemodynamic and respiratory components of CPR.

Electrical impedance tomography (EIT) is a noninvasive technique that measures lung ventilation at a high temporal resolution and its use is increasingly popular in the intensive care unit to set PEEP. To the best of our knowledge, except for an abstract^14^, there is no study about EIT during CPR published in full paper format. The present study was performed using fresh human cadavers aiming to explore whether the lung ventilation distribution measured by EIT was influenced by HUP during CPR. Our hypothesis is that lung ventilation should increase with HUP in particular in the dorsal lung regions provided that higher PEEP would not promote overdistension in the ventral lung regions and chest compressions would not decrease EELI.

## Methods

This was a prospective experimental cross-over study on human cadavers carried out in the anatomy laboratory. The human bodies were donated for medical science use by the donators themselves and warranting no ethical committee agreement (Additional File 1).

### Cadaver preparation

The trachea was intubated and connected to a ventilator (T60, Air Liquide Medical System, Antony, France) set in volume control mode, constant flow inflation profile, tidal volume 8 ml/kg predicted body weight (additional methods are available in the Additional File 1), respiratory rate 10 breaths/min, insufflation time 1 s, end-inspiratory pause 1 s, expiratory time 4 s, inspired oxygen fraction in air 21%, and PEEP 0 cmH_2_O. Airway pressure was measured at a heat-and-moisture exchanger (HME) (DAR, Covidien, Mansfield, MA) attached to the endotracheal tube. A flow-meter (Hamilton Medical, Switzerland) was inserted between the Y-piece and the HME. A nasogastric catheter (Nutrivent, Sidam, Mirandola, Italy) was inserted to measure oesophageal pressure. Flow, airway pressure and esophageal pressure signals were acquired at 200 Hz by a data-logger (Biopac 150, Biopac Inc., Golletta, CA). The ITD (ResQPOD ITD 16, Zoll Medical Corporation, Chelmsford, MA, USA) was inserted between the HME and the proximal tip of the endotracheal tube in part of the experiment. It generates a negative intrathoracic pressure at the time of chest decompression during CPR and hence increases the venous blood flow to the heart^15^.

### Cardiopulmonary resuscitation

CPR was performed by actively compressing and passively decompressing the chest with the Lund University Cardiopulmonary Assist Device (LUCAS 2, Stryker, USA) at a rate of 102/min. Chest compressions were not synchronized with the ventilator.

### Protocol

**Figure 1:**
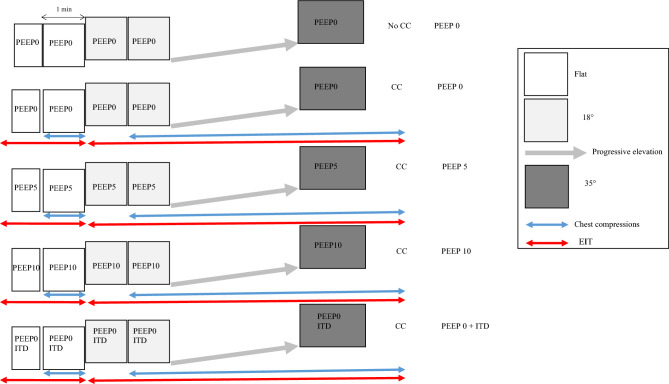
Schematic drawing of the sequences of events. *PEEP* positive end expiratory pressure, *ITD* impedance threshold device, *CC* chest compressions, *EIT* electrical impedance tomography.

Five sequences were randomly assigned to each cadaver according to a computer-generated table: no CPR-PEEP0, CPR-PEEP0, CPR-PEEP5, CPR-PEEP10, CPR-PEEP0-ITD (Fig. 1).

Each CPR sequence was performed across the same trunk inclination order: flat, 18° and 35°. When flat the cadaver was installed in a supine position with the back-plate under the body to which the LUCAS device was attached and locked with the suction cup over the chest. Before elevating the head and thorax the back-plate was replaced with one that provided an 18° inclination (EleGARD System, Advanced CPR Solutions, Mineapolis, MN, USA), to which the body was attached and locked with the LUCAS device, as in the flat step. When this second back plate was switched-on the body was gradually raised to the 35° inclination over 2 min, i.e. by an angle of 0.17°per second. Chest compressions were applied at the same angle to the chest as they were in the flat position.

In flat position chest compressions were performed over 1 min. During the 18–35° inclinations the chest compressions were started at 18° for 1 min, continued until the 35° inclination was reached and then continued for 90 s.

### Respiratory mechanics

Compliance of lung and chest wall was automatically measured breath by breath in the time without chest compressions in each trunk inclination, i.e. before and after chest compressions, via an application in Matlab (Matlab2021b, The Mathworks)^16^.

### Electrical impedance tomography

#### EIT device

A ring of 16 EKG electrodes (Blue sensor BR, Ambu®, Ballerup, Denmark) was placed around the thorax 5 cm above the xiphoid and connected to an EIT device (Gottingen High-Performance, Sensor Medics, Eindhoven, The Netherlands). A 5-mA alternating electrical current was applied and thorax scans were performed at 13.58 Hz.

#### EIT signal processing (Additional File 2).

**Figure 2:**
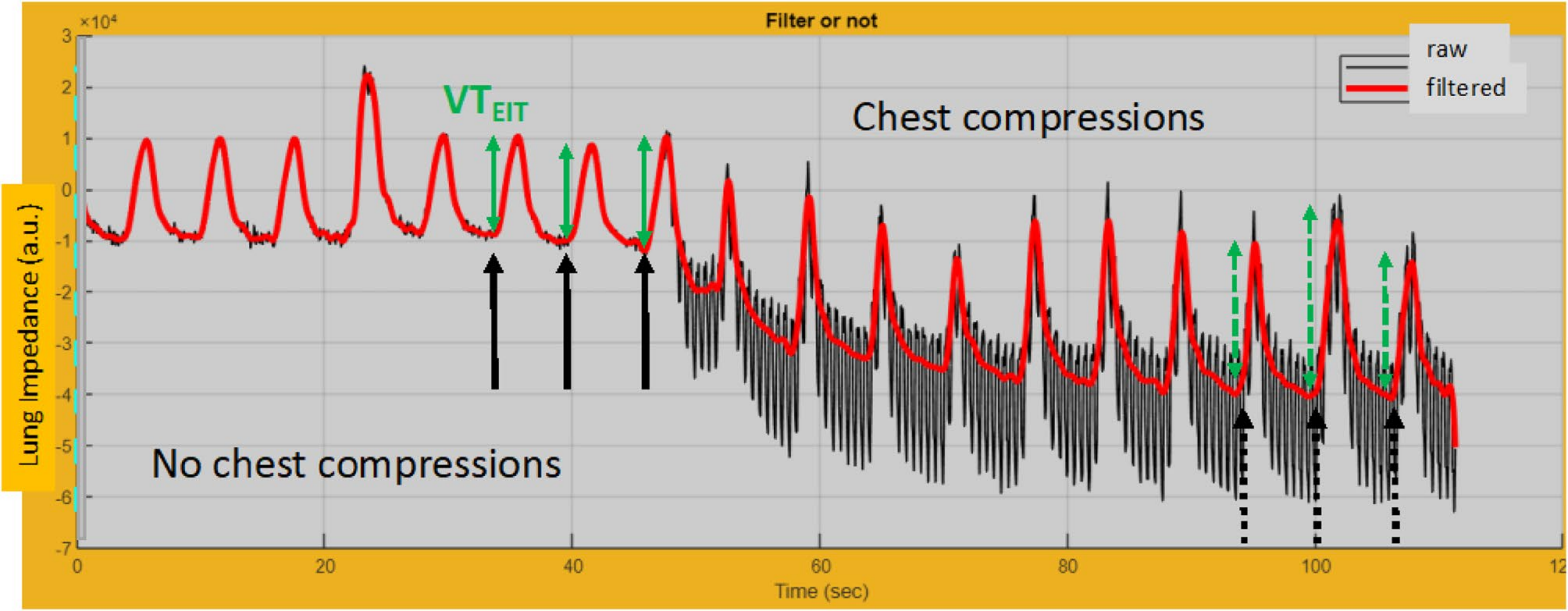
Time course of lung impedance (arbitrary units: a.u.) measured with electrical impedance tomography (EIT) in cadaver#6, (CPR-PEEP0 sequence, flat position) depicting the assessment of volume change (VT_EIT_) and end-expiratory lung impedance (EELI) before and after chest compressions. VT_EIT_ highlighted with the continuous and dotted double green arrows is the change between the minimal and the maximal values of the filtered lung impedance signal (red line). EELI is the minimal value. EELI was measured over the last three breaths before (continuous vertical black arrows) and during chest compressions once the tracing was stable (dotted vertical black arrows). EELI was normalized to VT_EIT_ of the corresponding breaths.

In each pixel and in the sum of all of them within a region of interest. The minimal and maximal values of the sum of all impedance pixel defined the onset and end, respectively, of inspiration (Fig. 2). The difference between them was termed VT_EIT_ because the changes in impedance correlates with the volume variations^17,18^.

Time course of lung impedance (arbitrary units: a.u.) measured with electrical impedance tomography (EIT) in cadaver#6, (CPR-PEEP0 sequence, flat position) depicting the assessment of volume change (VT_EIT_) and end-expiratory lung impedance (EELI) before and after chest compressions. VT_EIT_ highlighted with the continuous and dotted double green arrows is the change between the minimal and the maximal values of the filtered lung impedance signal (red line). EELI is the minimal value. EELI was measured over the last three breaths before (continuous vertical black arrows) and during chest compressions once the tracing was stable (dotted vertical black arrows). EELI was normalized to VT_EIT_ of the corresponding breaths.

#### EIT indexes

In addition to VT_EIT_, other EIT indexes based on the specific purpose of the study. The anterior-to-posterior VT_EIT_ ratio was determined for each region of interest. An anterior-to-posterior VT_EIT_ ratio of 1 reflected a balanced distribution of the ventilation between the dorsal and ventral lung regions. A ratio > 1 or < 1 indicated that most of the lung ventilation was distributed towards the anterior or the posterior lung regions, respectively. The pendelluft phenomenon was computed as the sum of all pixels belonging to regions of interest with negative VT_EIT_ differences. Pendelluft was normalized to the overall VT_EIT_ value and expressed as %VT_EIT_. Hence, as measured the pendelluft was in antiphase with the volume generated by the ventilator and thus pendelluft had a negative value. The pendelluft reflects an internal movement of gas during inspiration, from some parts of the lungs to other parts of the lung probably due to regional inequality in lung mechanics. This phenomenon, frequent in ICU patients^19^, is important because it is a mechanism by which dependent parts of the lung may get overdistended when they receive part of the inflated tidal volume from the non-dependent lung during the same inspiration^20^. The regional ventilation delay (RVD) measures the time delay to re-open atelectactic area with mechanical insufflation. It was computed according to Muders et al.^21^ on the EIT signal. In each pixel, the RVD was the percentage of the inflation time taken to reach 40% of the maximal value. An increase in the standard deviation of RVD (SDRVD) indicates a more heterogeneous distribution of regional inflation. The global inhomogeneity index (GII) measures the global spatial heterogeneity of ventilation. It was computed according to Zhao et al.^22^. The heterogeneity of ventilation increases with higher GII.

The end-expiratory lung impedance (EELI) was taken as the minimum value of lung impedance in each breath and normalized for VT_EIT_ (EELI/VT_EIT_) (Fig. 2).

#### EIT data analysis

**Figure 3:**
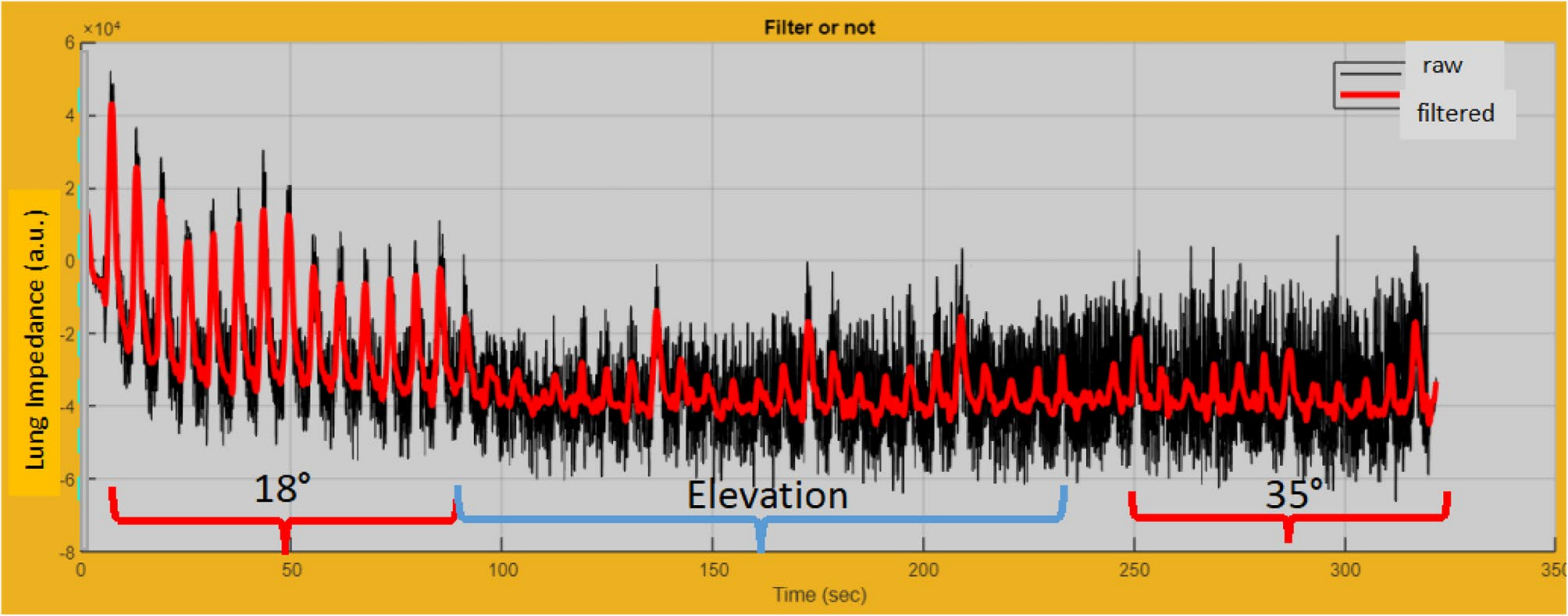
Time course of lung impedance (arbitrary units: a.u.) measured with electrical impedance tomography (EIT) in cadaver#6, (CPR- PEEP0 sequence, during elevation of the trunk from 18° to 35°).

VT_EIT_, anterior-to-posterior VTEIT ratio and pendelluft were measured on the same breaths over 60 s (10 cycles) during chest compressions in flat, 18° and 35° trunk inclinations. Figure 3 depicts the time course of lung impedance during elevation from 18° to 35° over which the EIT measurements were performed.

EELI and EELI/VT_EIT_ were measured over the last three breaths without and with cardiac compressions (Fig. 2).

### Statistical analysis

The primary end-point was VT_EIT_. The secondary end-points were: anterior-to-posterior distribution of VT_EIT_, pendelluft, EELI/VT_EIT_ and lung and chest wall compliance.

The analysis was conducted using a mixed-effects model for each sequence where the dependent variables were VT_EIT_, anterior-to-posterior VT_EIT_, pendelluft and EELI/VT_EIT_, the inclination (flat, 18°, 35°) the factor with fixed effect and the cadaver with the factor with a random effect . The reference position was flat, to which the two other inclinations were compared. The accuracy of the model was checked by plotting the standardized residuals to the fitted values.

Lung and chest all compliance were compared before and after cardiac compressions in flat position by using a mixed-effects model. They were also compared before cardiac compression at 18° versus after compression at 35° for the sequences 2–5. This was due to the fact that the chest compressions were continuously performed during elevation, and hence this comparison includes the effect of cardiac compression and of trunk inclination change as well. Values are presented as median (1st-3rd quartiles) unless otherwise stated.

### Ethics approval and consent to participate

The human bodies used in this study were donated for medical science use by the donators themselves. Written and witnessed consent to donate their bodies to science for anatomical and pedagogical purposes was given prior to death. This donation was free, anonymous, and regulated by the French funeral legislation. According to French law, no other approval was necessary by French authorities or by the local ethical board.

## Results

Seven cadavers (4 females and 3 males) of 89 ± 9 (mean ± SD) years of age and 51 ± 8 kgs predicted body weight were used (Additional File 3). Esophageal pressure was not available for one cadaver (failure to insert the nasogastric catheter in cadaver#1).

### Effect of inclination on VT_EIT_

**Figure 4:**
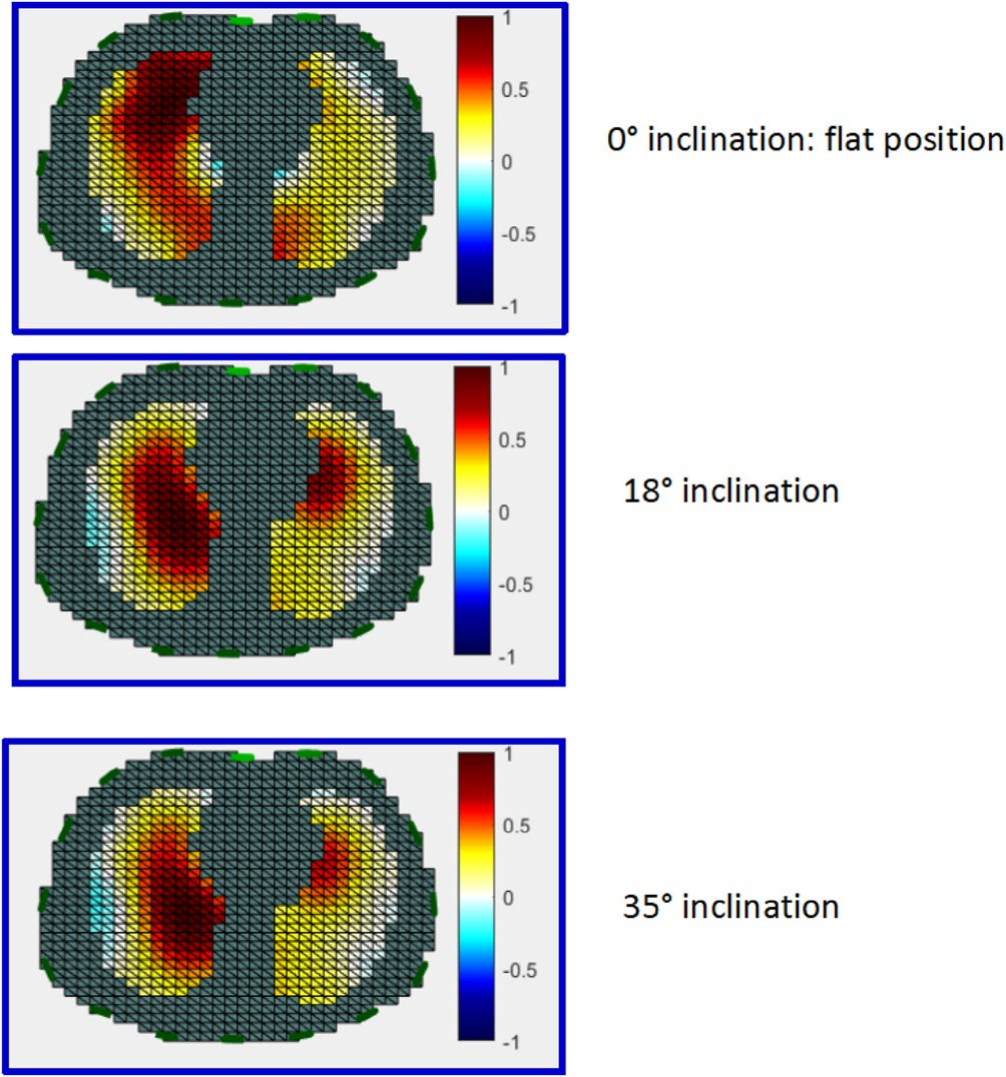
Parametric EIT images of tidal ventilation in cadaver#7 during cardiac compressions at different body inclinations. The blue areas show the pendelluft.

As shown in Table 1, the VT_EIT_ did not change significantly across the trunk inclination when the chest was not compressed. When chest compressions were performed VT_EIT_ decreased with trunk elevation as compared to flat position (Table 1). This decrease was consistently significant at 35° in every sequences with chest compressions and also at 18° in the CPR-PEEP0-ITD sequence. EIT parametric images of VT_EIT_ are shown in Fig. 4.

**Table 1:** Effect of body inclination on electrical impedance tomography indexes with or without chest compressions.

EIT index	Sequence	Flat	18° inclination	35° inclination
VT_EIT_ (a.u.)	No CPR-PEEP0	6597 (5130, 8878)	7348 (6702, 8822)	7010 (6901, 8194)
	CPR-PEEP0	12,416 (10,689, 14,442)	11,239 (7667, 13,292)	6457 (4631, 9516)*
	CPR-PEEP5	14,925 (12,425, 18,667)	14,791 (13,249, 17,194)	8960 (7434, 12,573)*
	CPR-PEEP10	14,481 (12,913, 17,110)	13,935 (11,857, 17,474)	9216 (5583, 11,758)*
	CPR-PEEP0-ITD	11,241 (9853, 16,933)	9900 (6153, 12,792)*	8211 (5602, 9645)*
VT_EIT_ Anterior-to-Posterior Ratio	No CPR-PEEP0	1.56 (1.08, 1.84)	1.87 (1.38, 2.10)	1.54 (0.80, 2.05)
	CPR-PEEP0	2.61 (1.57, 3.18)	2.16 (1.27, 2.54)	2.16 (1.30, 6.74)
	CPR-PEEP5	1.94 (1.72, 2.62)	2.07 (1.75, 2.34)	2.83 (1.96, 3.68)
	CPR-PEEP10	1.40 (1.05, 1.62)	1.48 (1.40, 1.88)	1.46 (1.20, 2.79)
	CPR-PEEP0-ITD	1.85 (1.34, 2.97)	1.86 (1.34, 3.17)	1.56 (0.79, 2.62)
Pendelluft (%VT_EIT_)	No CPR-PEEP0	− 12 (− 22, − 6)	− 4 (− 5, − 3)*	− 5 (− 9, − 3)
	CPR-PEEP0	− 9 (− 15, − 4)	− 6 (− 22, − 2)	− 6 (− 28, − 2)
	CPR-PEEP5	− 7 (− 10, − 3)	− 4 (− 10, − 2)	− 7 (− 10, 3)
	CPR-PEEP10	− 3 (− 4, − 3)	− 3 (− 6, − 3)	− 5 (− 7, − 5)
	CPR-PEEP0-ITD	− 7 (− 11, − 3)	− 8 (− 11, − 4)	− 11 (− 16, − 9)
EELI/VT_EIT_	No CPR-PEEP0	− 0.46 (− 0.47, − 0.40)	− 0.30 (− 0.54, − 0.27)	− 0.38 (− 1.18, − 0.32)
	CPR-PEEP0	− 0.61 (− 1.43, − 0.49)	− 0.63 (− 1.08, − 0.06)	− 1.31 (− 2.98, − 0.80)
	CPR-PEEP5	− 1.15 (− 1.38, − 0.90)	− 0.81 (− 1.15, − 0.59)	− 1.41 (− 2.33, − 0.32)
	CPR-PEEP10	− 1.00 (− 1.23, − 0.70)	− 1.38 (− 1.78, − 0.98)	− 2.80 (− 3.00, − 2.24)*
	CPR-PEEP0-ITD	− 0.76 (− 1.16, − 0.32)	− 0.37 (− 0.78, − 0.15)	− 1.15 (− 1.56, − 0.15)
SDRVD (% inspiratory time)	No CPR-PEEP0	4.15 (4.03,4.57)	3.35 (2.92, 4.15)	3.00 (2.89, 3.31)
	CPR-PEEP0	4.96 (4.45,6.66)	4.80 (3.35, 5.66)	3.66 (3.16, 4.36)
	CPR-PEEP5	4.13 (3.64, 7.23)	4.47 (3.38, 6.61)	3.15 (2.77, 3.58)
	CPR-PEEP10	4.17 (3.85, 6.92)	6.86 (3.95, 10.06)	3.31 (3.11, 4.12)
	CPR-PEEP0-ITD	4.44 (4.09, 5.82)	6.83 (3.95, 8.41)	3.72 (3.13, 3.93)
GII	No CPR-PEEP0	0.98 (0.73, 1.22)	0.73 (0.69, 0.81)	0.76 (0.67, 0.86)
	CPR-PEEP0	0.75 (0.59, 0.94)	0.71 (0.63, 0.72)	0.71 (0.66, 0.94)
	CPR-PEEP5	0.63 (0.59, 0.76)	0.69 (0.66, 0.77)	0.80 (0.61, 0.84)
	CPR-PEEP10	0.56 (0.55, 0.61)	0.63 (0.56, 0.68)	0.70 (0.58, 0.85)
	CPR-PEEP0-ITD	0.80 (0.66, 0.88)	0.85 (0.65, 0.89)	0.88 (0.66, 0.91)

Values are median (1st, 3rd quartiles).

*EIT* electrical impedance tomography, *VT*_EIT_ tidal ventilation, *EELI* end-expiratory lung impedance, *a.u*. arbitrary units, *CPR* cardiopulmonary resuscitation, *PEEP* positive end-expiratory pressure, *ITD* impedance threshold device, *SDRVD* standard deviation of the regional ventilation delay, *GII* global inhomogeneity index.

*P < 0.05 vs. Flat.

### Effect of inclination on anterior-to-posterior VT_EIT_, pendelluft and EELI/VT_EIT_, SDRVD and GII

The anterior-to-posterior VT_EIT_ did not statistically significantly change with trunk elevation in any sequence (Table 1). The same was true for pendelluft except that it was lower at 18° in the sequence 1 without chest compression. EELI/VT_EIT_ became more negative, i.e. EELI decreased, with trunk elevation in every sequence but the first, the difference being statistically significant only for the CPR-PEEP10 sequence (Table 1). There was a non-significant trend to a reduction in SDRVD with body inclination in every sequence (Table 1). With body inclination GII non-significantly tended to decrease in absence of chest compression and at PEEP 0 and to increase in the other conditions (Table 1).

### Effect of chest compressions on EELI/VT_EIT_

**Figure 5:**
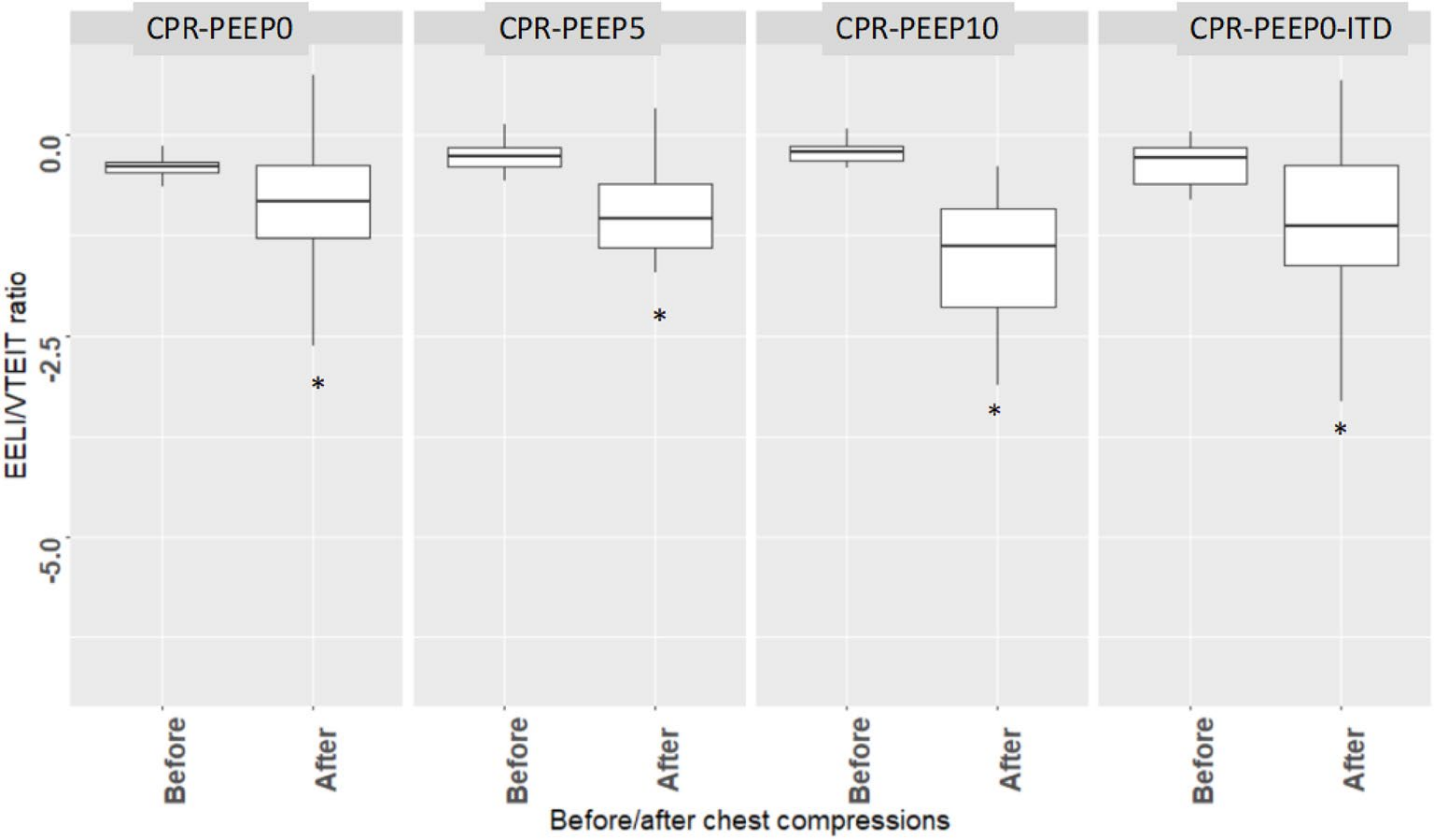
Box-and-whisker plots of end expiratory lung impedance to change in impedance (EELI/VT_EIT_) ratio before and after chest compressions during the 4 sequences with chest compressions. *CPR* cardiopulmonary resuscitation, *PEEP* positive end-expiratory pressure, *ITD* impedance threshold device. *P < 0.05 vs before.

After chest compressions EELI/VT_EIT_ was significantly lower than before chest compressions in every sequence with CPR (Fig. 5). Across all of them it significantly decreased from − 0.30 (− 0.40; − 0.15) before to − 1.13 (− 1.70; − 0.61) after the chest compressions (P = 0.009).

### Effect of chest compressions on lung and chest wall compliance

In the flat position the lung and chest wall compliance increased after chest compressions except in the CPR-PEEP0-ITD sequence for the lung compliance and in the CPR-PEEP5 sequence for the chest wall (Table 2). During trunk elevation, the chest wall and lung compliances were higher after than before chest compressions, expect for lung compliance, which was lower in sequence CPR-PEEP5 and also in CPR-PEEP0-ITD sequence though not significantly.

**Table 2:** Change in lung and chest wall compliance with chest compressions.

Sequence	Position	Change in lung compliance	Change in chest wall compliance
CPR-PEEP0	Flat	+ 6.2 [+ 5.2, + 7.1]**	+ 26.4 [+ 14.3, + 38.5]**
CPR-PEEP5	Flat	+ 5.8 [+ 4.9, + 6.7]**	+ 14.1 [− 30.0, + 58.2]
CPR-PEEP10	Flat	+ 2.6 [+ 0.3, + 4.8]**	+ 112.2 [+ 77.7, 147.8]**
CPR-PEEP0-ITD	Flat	− 2.3 [− 3.7, − 0.9]**	+ 8.7 [+ 6.1, + 11.2]**
CPR-PEEP0	Elevation	+ 5.6 [+ 0.6, + 10.5]*	+ 94.7 [+ 15.7, + 174.1]*
CPR-PEEP5	Elevation	− 4.7 [− 8.8, − 0.7]*	+ 37.0 [+ 17.0, + 57.1]**
CPR-PEEP10	Elevation	+ 5.1 [+ 2.5, + 7.7]*	+ 86.7 [+ 23.8, + 149.6]*
CPR-PEEP0-ITD	Elevation	− 2.5 [− 6.7, + 1.9]	+ 23.2 [+ 12.1, + 34.2]**

Values are mean [95% confidence intervals] ml/cmH_2_O. Lung and chest wall compliance are measured without chest compressions, i.e. immediately before and after them. The change is the absolute difference between after and before chest compressions, a positive value indicating that the compliance is greater after than before chest compressions and the opposite is true for a negative value. Elevation means trunk angulation moved from 18° to 35°. The change in compliance reflects both the effect of chest compression and the change in trunk inclination.

*CPR* cardiopulmonary resuscitation, *PEEP* positive end-expiratory pressure, *ITD* impedance threshold device.

*P < 0.05, **P < 0.001.

## Discussion

To our knowledge, this is the first study that measures the distribution of lung ventilation with EIT during flat and HUP-CPR in human cadavers. The main findings were: (1) with HUP-CPR, VT_EIT_ decreased at PEEP values of 0, 5 and 10 cmH_2_O compared to flat CPR, but its anterior-to-posterior distribution did not significantly change; the same was found with ITD in place; (2) the chest compressions decreased EELI/VT_EIT_.

### Methodology

Before to discuss present results, a critique of our methodology is required. The study challenged the EIT technology in many ways. One was the location of the electrodes across the thorax. Using a ring of electrodes rather than a belt was thought more flexible to accommodate the LUCAS installation. Another issue was the baseline recording. EIT depends on a measurement of change in impedance relative to a baseline. Performing a continuous EIT recording that would have taken more than one hour had several risks. One was a potential electrode contact defect during the different maneuvers and the loss of parts of the records. Therefore, the design took into account this issue and the experiment was split into 5 sequences for which the baseline was as close as possible to the recording of the head elevation maneuver. Another risk was the skin contact of the electrodes. To manage it, the EIT device continuously monitored the electrode impedance in order to maintain it in the appropriate range. Finally, the issue of filtering the EIT signal disturbed by chest compression artefacts was solved as shown in Figs. 1, 2 giving a good signal-to-noise ratio. Furthermore, the accuracy of filtering was tested after each recording with our flexible application developed in Matlab. Contrary to most of the commercial EIT systems with which the sternum and spine are avoided with electrodes placed to inject more electric current into the chest cavity, our device included one sternal and one spinal electrode. As we argued in the Additional File 2, we decided not to use a conventional EIT belt due to the presence of the CPR device placed in the mid-sternum. We reasoned that by using a series of 16 EIT electrodes would facilitate the management of the CPR with that device. It is true that placing the electrodes 1 and 9 at the sternum and spine, respectively, was the way the EIT prototype was used and published in many publications about EIT^18,23–25^ before the new commercial devices came up. To the best of knowledge, there was no comparison between the single 16 electrodes and the 16-electrodes belt.

### Model validation

The present study shows that EIT is feasible during CPR in humans, which has clinical and research implications. Beyond this, we are aware that it is important to make sure that the present results are consistent with past and future findings.

Two findings deserve attention in the perspective of a model validation. Without chest compressions at PEEP0, EELI/VT_EIT_ did not change with HUP in present study. By contrast, in acute respiratory distress syndrome patients HUP was associated with, on one hand, an increased EELV^26–29^, and, on the other hand, higher plateau pressure and lower respiratory system compliance that improved with chest strapping^30^. Both findings suggest that HUP may induce end-inspiratory overdistension and that an increase in EELV may reflect overdistension. The anterior-to-posterior distribution of VT_EIT_ was markedly greater than 1 in the flat position in all the sequences, indicating a marked distribution of tidal volume towards the most anterior parts of the lung during mechanical ventilation and CPR. This may also reflect atelectasis in the dorsal lung regions.

### Effect of HUP and chest compressions on VT_EIT_ at different PEEP values

In present study we measured VT_EIT_, its anterior-to-posterior distribution and the pendelluft during chest compressions at various trunk inclinations. In the flat position, VT_EIT_ was twice higher with CPR at any PEEP than without at PEEP0 (Table 1). We did not formally compare these values because the baseline may differ across the sequences. VT_EIT_ decreased with HUP consistently across all PEEP levels. This finding may reflect overdistension with HUP, in particular in the ventral lung regions, or by collapse in the dorsal lung regions or by a combination of both. Our study design did not permit to further explore these mechanisms. Ascribing the findings entirely to ventral overdistension might be too simplistic, in particular because the VT_EIT_ distribution did not change between conditions. The fact that GII increased, though not significantly, with body inclination at PEEP 5 and 10 when RVD decreases may suggest overinflation in the ventral regions, should it be assumed that the lower SDRDV reflects recruitment in the dorsal lung regions. It should be noted, however, that there were changes in that distribution but these were not statistically significant perhaps by lack of power. However, the anterior-to-posterior distribution of VT_EIT_ did not change significantly with HUP as compared to baseline. With the ventral lung regions overdistended, the ventilation in those regions, and hence the anterior-to-posterior of VT_EIT_ would likely be lower with HUP. On the other hand, the pendelluft phenomenon, which was present in the flat position in each sequence, did not significantly change with HUP during the chest compressions.

Yang et al. measured some EIT indexes in 30 normal volunteers breathing spontaneously at 0, 30, 60 and 90 degrees from the horizontal plane in the supine position^31^. They found that with the sitting positioning the dorsal ventilation increased and the ventilation became more heterogeneous. Moreover, the authors had concern about keeping the same the position of the electrodes over the range of inclination. Our results are difficult to compare with that of Yang et al. given the difference in subjects and ventilation regimen^31^.

### Effect of chest compressions on EELI/VT_EIT_

We measured EELI/VT_EIT_ before and after chest compressions at different trunk angulations and PEEP values. The present study confirms that chest compressions during non-synchronous mechanical ventilation decreases EELI, as already reported for EIT in an experimental model of cardiac arrest in pigs^14^ and in other studies^32,33^. This also argues in favor of the model’s validity. The clinical implication for this finding would be to set some PEEP during the chest compressions or once the circulation is restored in order to optimize ventilation, the balance with the effect on cerebral perfusion and venous return is still to be determined. However, in present study even with PEEP, EELI/VT_EIT_ decreased with HUP, suggesting that HUP would not improve lung ventilation.

### Lung and chest wall mechanics

In human cadavers a previous study found that the chest wall compliance increased over time after chest compressions^34^. In present study we assessed the chest wall compliance by using esophageal manometry and also found it was higher after than before chest compressions, in each trunk inclination and at each PEEP value. The chest wall was softened by the prolonged chest compression. This confirmative finding is another evidence validating our experimental model. In the absence of lung CT scans it was not possible to assess the nature and extent of lung injury, if any, in the cadavers we used. It is, however, likely that the bodies had some degree of lung edema and or tissue edema.

### Limitations

Our study has several limitations. One is the use of a cadaver model. For this reason, no recommendation can be made regarding inclination or the use if an ITD because no information about hemodynamics and cerebral perfusion could be collected during present experiment. Tidal volume measured by EIT does not account for the presence of pulmonary ventilation due to chest compressions, which may also be influenced by chest elevation and PEEP. As mentioned above, the lack of lung CT to better define the presence and extent of lung injury at baseline is a limitation. Our study is a pilot study and probably underpowered due to the cost and availability of cadavers. Therefore, it only allows us to track trends and we cannot draw any firm conclusions. A better understanding of the effects of chest compressions and the impact of mechanical ventilation during CPR on lung ventilation distribution could impact the use of mechanical ventilation after cardiac arrest^35^.

## Conclusions

HUP during chest compressions decreased lung ventilation compared to standard flat CPR. This reduction might be linked with the reduction in pulmonary overdistension during CPR or simply a decrease below normal values. Chest compressions decreased end-expiratory lung impedance.

### Supplementary Information

Supplementary Information 1.

Supplementary Information 2.

Supplementary Table 1.

## Data Availability

All data generated or analysed during this study are included in this published article [and its supplementary information files].

## Abbreviations

CPR: Cardiopulmonary resuscitation
EELV: End-expiratory lung volume
PEEP: Positive end-expiratory pressure
ITD: Impedance threshold device
HUP: Head-up
EIT: Electrical impedance tomography
HME: Heat and moisture exchanger
LUCAS: Lund University Cardiopulmonary Assist
VTEIT: Change in lung impedance during breathing cycles
EELI: End-expiratory lung impedance
GII: Global inhomogeneity index
SDRVD: Standard deviation of regional ventilation delay
