# Supplementary material for: Effect of automated head-thorax elevation during chest compressions on lung ventilation: a model study
Source: Sci Rep. 2023 Nov 21;13:20393. doi: 10.1038/s41598-023-47727-z (PMC10663599; doi:10.1038/s41598-023-47727-z)
Supplement: Supplementary file 1 — Supplementary Information 1. [file 41598_2023_47727_MOESM1_ESM.docx]

**Additional File 1**

**Methods**

*Ethical Statement*

The human bodies used in this study were donated for medical science use by the donators themselves. Written and witnessed consent to donate their bodies to science for anatomical and pedagogical purposes was given prior to death. This donation was free, anonymous, and regulated by the French funeral legislation. According to French law, no other approval was necessary by French authorities or by the local ethical board.

*Cadaver preparation*

Cadavers were frozen and preserved at -22°C as soon as they arrived at the laboratory. Bodies were thawed at ambient temperature four days before the start of each experiment. The blood was not washed out.

*Ventilator check*

The ventilator was calibrated before each experiment and the same double-limb ventilator circuit was used throughout the experiment (Intersurgical Ltd, Berkshire, UK).

*Predicted Body weight calculation*

The PBW was equal to 50+0.91 x (height in centimeters -152.4) for males and 45.5+0.91 x (height in centimeters -152.4) for females.
