# Supplementary material for: Effect of automated head-thorax elevation during chest compressions on lung ventilation: a model study
Source: Sci Rep. 2023 Nov 21;13:20393. doi: 10.1038/s41598-023-47727-z (PMC10663599; doi:10.1038/s41598-023-47727-z)
Supplement: Supplementary file 2 — Supplementary Information 2. [file 41598_2023_47727_MOESM2_ESM.docx]

**Additional File 2**

**Electrical Impedance Tomography**

Electrical Impedance Tomography device

For this experiment we decided not to use a belt but rather individual electrodes to accommodate the presence of the suction cup of the mechanical CPR device on the sternum. A ring of 16 EKG electrodes (Blue sensor BR, Ambu®, Ballerup, Denmark) was placed around the thorax 5 cm above the xiphoid and connected to an EIT device (Gottingen High-Performance, Sensor Medics, Eindhoven, The Netherlands). The electrode #1 was placed just below the suction cup and electrodes # 2 and 16 to the left and right of it, respectively (Figure S1). Therefore, apart from electrode#1, the 15 electrodes were in the same plane across the thorax. A 5-mA alternating electrical current was applied and thorax scans were performed at 13.58 Hz.


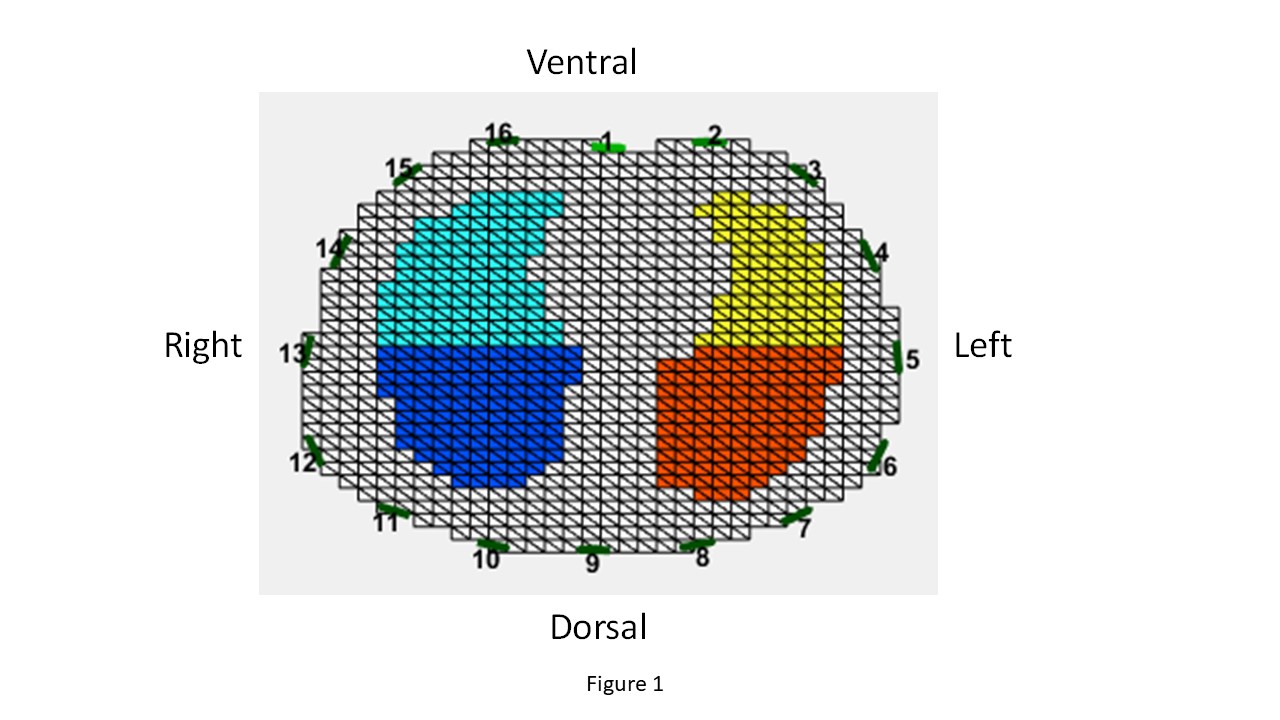


Figure A1. Electrode positions (green lines) and regions of interest in the 4 quadrants (cyan, blue, yellow and orange regions). Cyan and yellow regions define the anterior parts of the lungs while the blue and orange regions define the posterior parts.

Electrical Impedance Tomography signal processing

The EIT signals were processed with diffuse optical tomography reconstruction software (EIDORS) ^1^ licensed under a general public GNU (http://eidors3d.sourceforge.net/) associated with the MATLAB scripting language. In this reconstruction, we used the “adult_male_16el” GREIT model ^2^. This model was also used to define the regions of interest (ROI) within the chest contours of the right and left lung, divided into four quadrants (figure S1).

To reduce artefacts due to chest compressions a 12th-order Butterworth low-pass filter (3-dB, 1.05 Hz) was applied to the EIT data. Once the sequence of raw EIT images was built (32 x 32 pixels, 13.58 images/s), the EIT waveform (overall impedance change as a function of time) was generated by summing the value of all pixels over the ROI in each image.

References

1. Adler A, Lionheart WR. Uses and abuses of EIDORS: an extensible software base for EIT. Physiol Meas 2006;27:S25-42.

2. Adler A, Arnold JH, Bayford R, et al. GREIT: a unified approach to 2D linear EIT reconstruction of lung images. Physiol Meas 2009;30:S35-55.
