# Supplementary material for: Effect of automated head-thorax elevation during chest compressions on lung ventilation: a model study
Source: Sci Rep. 2023 Nov 21;13:20393. doi: 10.1038/s41598-023-47727-z (PMC10663599; doi:10.1038/s41598-023-47727-z)
Supplement: Supplementary file 3 — Supplementary Table 1. [file 41598_2023_47727_MOESM3_ESM.docx]

**Additional file 3**

Table 1. Characteristics of the cadavers used in present study

| Cadaver | Sex | Age (year) | Weight (kg) | Height (cm) | BMI (kg/m^2^) | Days of death to present study |
| --- | --- | --- | --- | --- | --- | --- |
| 1 | Male | 93 | 60 | 163 | 23 | 32 |
| 2 | Female | 70 | 60 | 151 | 26 | 79 |
| 3 | Male | 94 | 75 | 165 | 28 | 41 |
| 4 | Female | 93 | 45 | 145 | 21 | 10 |
| 5 | Male | 88 | 35 | 153 | 15 | 24 |
| 6 | Male | 92 | 50 | 163 | 19 | 7 |
| 7 | Female | 90 | 55 | 152 | 24 | 7 |
| Mean |  | 89 | 54 | 156 | 22 | 29 |
| SD |  | 8 | 12 | 7 | 4 | 24 |

BMI: body mass index, SD: standard deviation

Cadavers were frozen and preserved at -22°C as soon as they arrived at the laboratory. Bodies were thawed at ambient temperature four days before the start of each experiment.
